# ^1^H NMR-based Investigation of Metabolic Response to Electro-Acupuncture Stimulation

**DOI:** 10.1038/s41598-017-07306-5

**Published:** 2017-07-28

**Authors:** Caigui Lin, Zhiliang Wei, Kian-Kai Cheng, Jingjing Xu, Guiping Shen, Chang She, Huan Zhong, Xiaorong Chang, Jiyang Dong

**Affiliations:** 1 Department of Electronic Science, Fujian Provincial Key Laboratory for Plasma and Magnetic Resonance, Xiamen University, Xiamen, 361005 China; 2 Department of Radiology, The Johns Hopkins University, Baltimore, Maryland 21205 USA; 3 Department of Bioprocess & Polymer Engineering and Innovation Centre in Agritechnology, Universiti Teknologi Malaysia, 81310 Johor, Malaysia; 4 College of Acupuncture & Moxibustion and Tui-na, Hunan University of Chinese Medicine, Changsha, 410007 China

## Abstract

Acupuncture is a traditional Chinese medicine therapy that has been found useful for treating various diseases. The treatments involve the insertion of fine needles at acupoints along specific meridians (meridian specificity). This study aims to investigate the metabolic basis of meridian specificity using proton nuclear magnetic resonance (^1^H NMR)-based metabolomics. Electro-acupuncture (EA) stimulations were performed at acupoints of either Stomach Meridian of Foot-Yangming (SMFY) or Gallbladder Meridian of Foot-Shaoyang (GMFS) in healthy male *Sprague Dawley* (SD) rats. ^1^H-NMR spectra datasets of serum, urine, cortex, and stomach tissue extracts from the rats were analysed by multivariate statistical analysis to investigate metabolic perturbations due to EA treatments at different meridians. EA treatment on either the SMFY or GMFS acupoints induced significant variations in 31 metabolites, e.g., amino acids, organic acids, choline esters and glucose. Moreover, a few meridian-specific metabolic changes were found for EA stimulations on the SMFY or GMFS acupoints. Our study demonstrated significant metabolic differences in response to EA stimulations on acupoints of SMFY and GMFS meridians. These results validate the hypothesis that meridian specificity in acupuncture is detectable in the metabolome and demonstrate the feasibility and effectiveness of a metabolomics approach in understanding the mechanism of acupuncture.

## Introduction

Acupuncture is a traditional Chinese medicine therapy that has been practised in China for 4,000 years^1^. More recently, it has emerged as a complementary therapeutic method for health restoration, promotion, and maintenance^1, 2^. The practice of acupuncture involves the insertion of fine needles into acupuncture points (acupoints) in the body. To date, more than 400 acupoints affiliated to 12 meridians across the human body have been systemically described. Application of acupuncture on a specific median exerts a therapeutic effect on a specific part of the human body. This specific effect, known as meridian specificity, forms the theoretical basis of acupuncture therapy^3^. Researchers have reported the existence of meridian specificity with functional neuroimaging techniques^4–7^.

With the aid of modern clinical and experimental techniques, there is increasing evidence regarding the efficacy of acupuncture in ameliorating symptoms of diseases, such as gastroesophageal reflux disease^8^, functional dyspepsia^9^, insomnia^10^, migraines^11^, and rheumatoid arthritis^12^. However, the biological mechanism underlying how acupuncture actually works remains elusive.

Metabolomics serves as an emerging tool with rapid expansion for high-throughput biomedical analyses^13^ and provides a “snapshot” of the metabolic status of a biological sample^14, 15^. Interest in applying NMR-based metabolomics to study the metabolic changes following acupuncture treatment is increasing^16–20^. In this study, we used a proton nuclear magnetic resonance- (^1^H NMR-) based metabolomics approach to investigate the metabolic perturbations due to acupuncture at two different specific meridians, namely, the Stomach Meridian of Foot-Yangming (SMFY) and Gallbladder Meridian of Foot-Shaoyang (GMFS) meridians. According to previous reports, electro-acupuncture (EA) treatment on acupoints along the SMFY meridian proved effective in enhancing gastrointestinal motility, improving gastric mucosal blood flow, and protecting gastric mucosa from injury^21–23^. On the other hand, EA treatment on acupoints in the GMFS meridian was found to promote bile production, secretion, and release to facilitate digestion, improve insomnia and to alleviate migraines^24, 25^.

**Figure 1:**
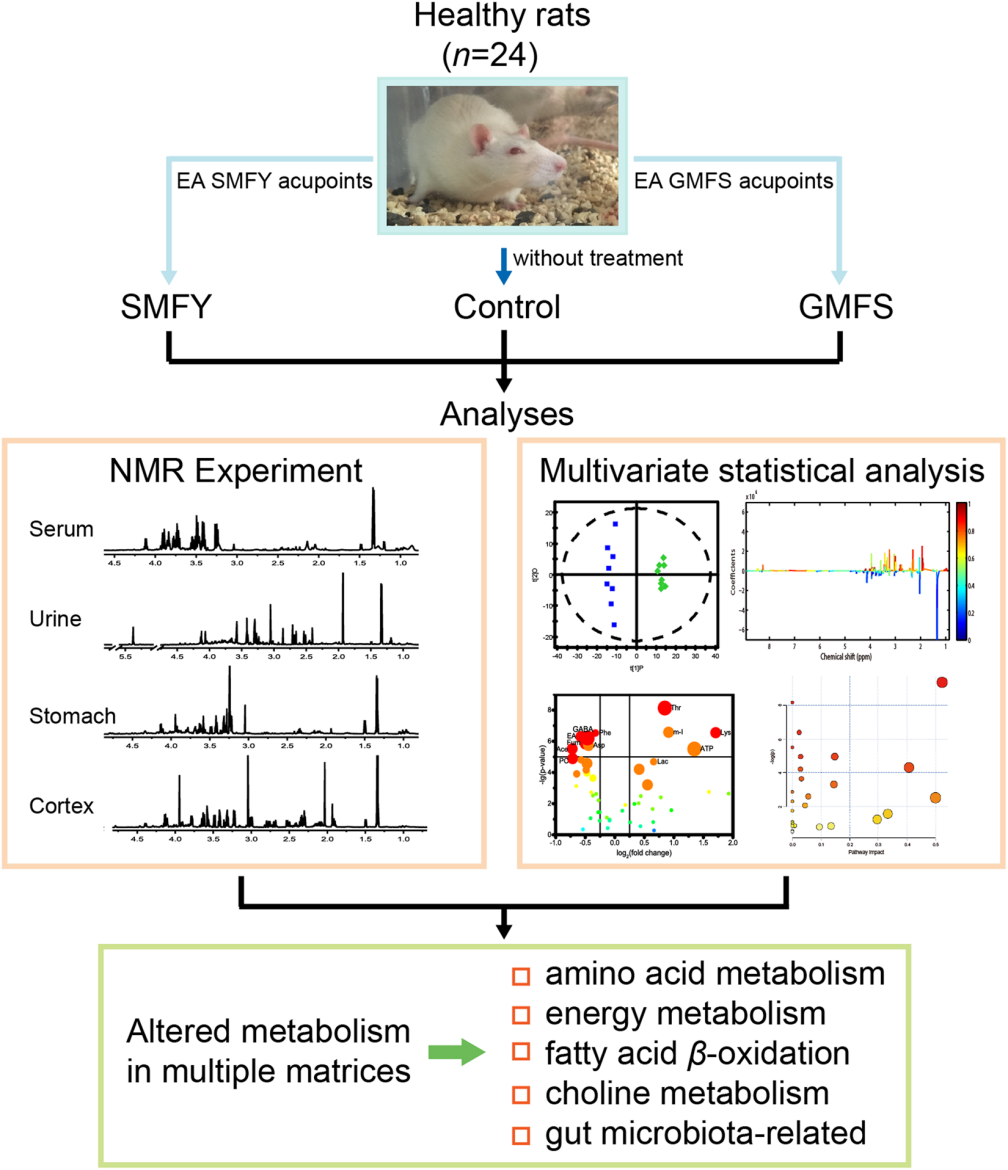
Flowchart of experimental design and analyses. Twenty-four healthy SD rats were divided into three groups: control, SMFY and GMFS groups. Samples of serum, urine, stomach and cortex were measured with ^1^H NMR spectroscopy to examine altered metabolism due to acupuncture treatments.

The flow chart of experiments in this study is shown as Fig. 1. Our primary purpose is to validate the hypothesis that meridian specificity in acupuncture is detectable in the metabolome and to provide specific metabolic patterns induced by acupuncture stimuli on different meridians.

Assignments: 2-HB, 2-hydroxybutyrate; 3-HB, 3-hydroxybutyrate; Ace, acetate; Ade, adenosine; AH, aminohippurate; Ala, alanine; All, allantion; Asp, asparate; Ben, benzoate; Bet, betaine; Ci, citrate; Cn, creatinine; Cr, creatine; DMA, dimethylamine; DMG, *N,N*-dimethylglycine; EthA, ethanolamine; For, formate; GABA, *γ*-aminobutyrate; Gln, glutamine; Glu, glutamate; Gly, glycine; GPC, glycerophosphocholine; Hip, hippurate; HX, hypoxanthine; Ino, inosine; Lac, lactate; LDL, low density lipoprotein; Lys, lysine; Met, methionine; *m*-I, *myo*-inositol; MM, methylmalonate; MN, *N*-methylnicotinamide; *m*-HPA, *meta*-hydroxyphenylacetate; NAA, *N*-acetylaspartate; OAS, *O*-acetylglycoprotein; *o*-HPA, *ortho*-hydroxyphenylacetate; PAG, phenylacetylglycine; PC, phosphocholine; Phe, phenylalanine; Tau, taurine; Thr, threonine; Tyr, tyrosine; Uc, urocanate; Ura, uracil; *α*-Glc, *α*-glucose; *α*-KG, *α-*ketoglutarate; *β*-Glc, *β*-glucose.

## Results

### ^1^H NMR spectra of multiple biological samples

**Figure 2:**
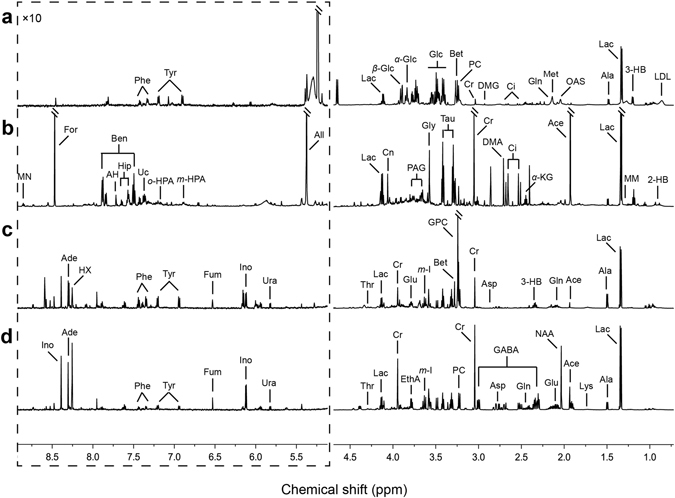
Typical ^1^H NMR spectra (at 600 MHz) of multiple matrices in the control group. (**a**) serum, (**b**) urine, (**c**) stomach extract, (**d**) cortex extract.

Typical ^1^H NMR spectra for the control group are shown as Fig. 2. In the figure, high-intensity peaks are assigned based on published literature^26^ and the HMDB database (http://www.hmdb.ca/). Generally, the spectra show common peaks (which are present in different types of samples) including choline, purine, amino acids, carboxylic acids, and glycolysis and TCA cycle intermediates. Some of the metabolites are tissue-specific; for example, cortex tissue extract contains neurotransmitter (GABA), serum contains lipoprotein and glycoprotein, and urinary samples present gut microbiota-related metabolites, such as hippurate, benzoate, and urocanate.

### Pattern recognition analyses

**Figure 3:**
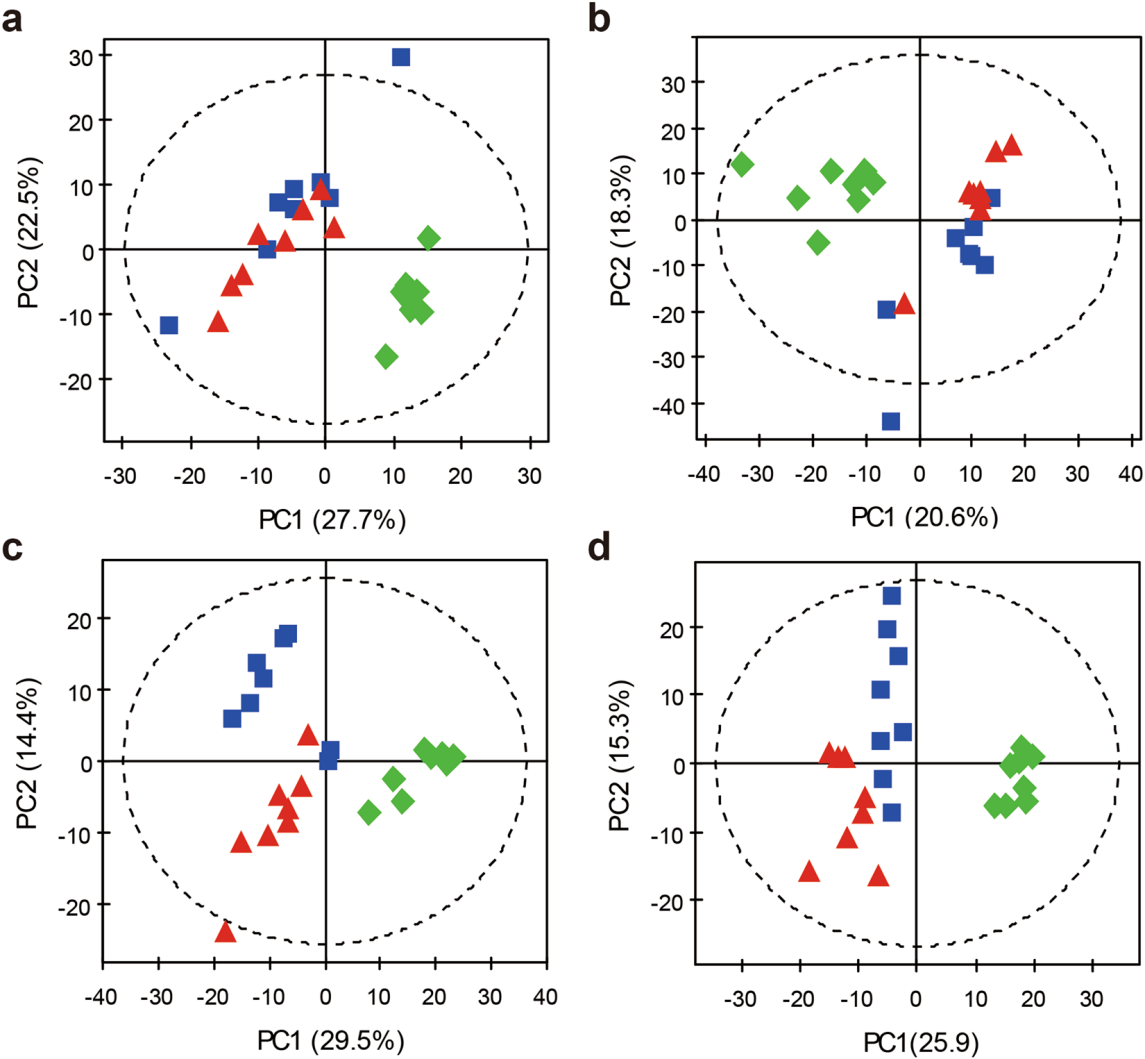
PCA scores plots of the control (), SMFY () and GMFS () groups for (**a**) serum, (**b**) urine, (**c**) stomach extract, and (**d**) cortex extract.

Next, we used multivariate statistical analyses to examine the metabolic differences between EA stimulations on the SMFY and GMFS meridians. Analysis of the NMR data using PCA shows good separation between the control group and EA-treated groups (i.e., SMFY and GMFS) for all types of biological samples (Figs 3 and S1). The result showed that EA treatment contributed most to the metabolic variation among the groups.

**Figure 4:**
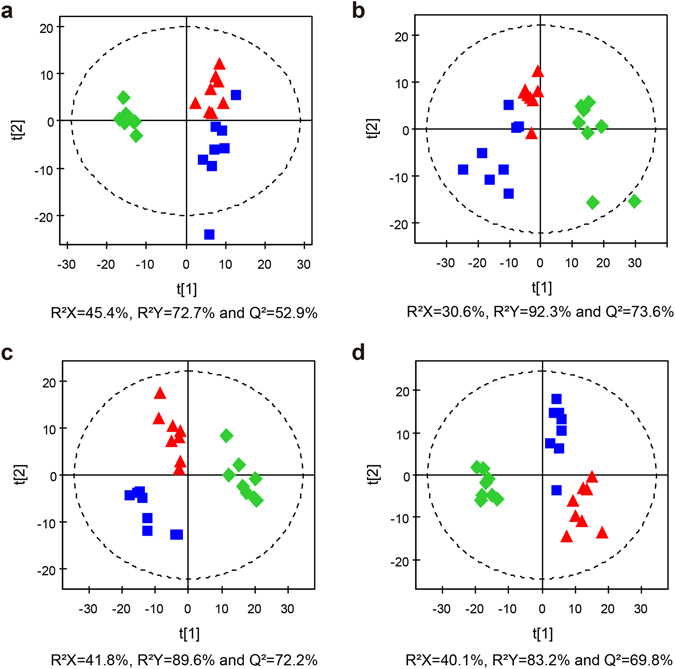
PLS-DA scores plots of the control (), SMFY () and GMFS () groups for (**a**) serum, (**b**) urine, (**c**) stomach extract, and (**d**) cortex extract.

PLS-DA modelling was further used to examine class discrimination. In addition to group separation between the controls and EA-stimulated rats, the PLS-DA scores plots further showed good separation between the SMFY and the GMFS groups (Fig. 4). The result suggested that the meridian-specific metabolic changes due to acupuncture are detectable in the metabolome of biological samples. In particular, both EA-treated groups (i.e., SMFY and GMFS) were well separated from the control group along the first latent component (t[1]), indicating that metabolic profiles of rat bio-samples were distinctly altered by the EA stimulation. Apart from the separation by t[1], the second latent component (t[2]) further showed separation between the SMFY and GMFS groups. Furthermore, comparisons between the control and the two treatment groups (i.e., control and SMFY, control and GMFS) were carried out using the PLS-DA and OPLS-DA analyses. The models were found robust following a 7-fold cross-validation and permutation test (200 permutations) (Figs S2 and S3).

We will next discuss two aspects of the metabolic analyses: first, a comparison between the EA-stimulated and the control groups to investigate the non-specific metabolic responses due to EA stimulation (meridian independent), and second, a comparison between the SMFY and GMFS groups to investigate the specific metabolic perturbation due to EA stimulations on different meridians.

### EA-perturbed metabolic changes

**Figure 5:**
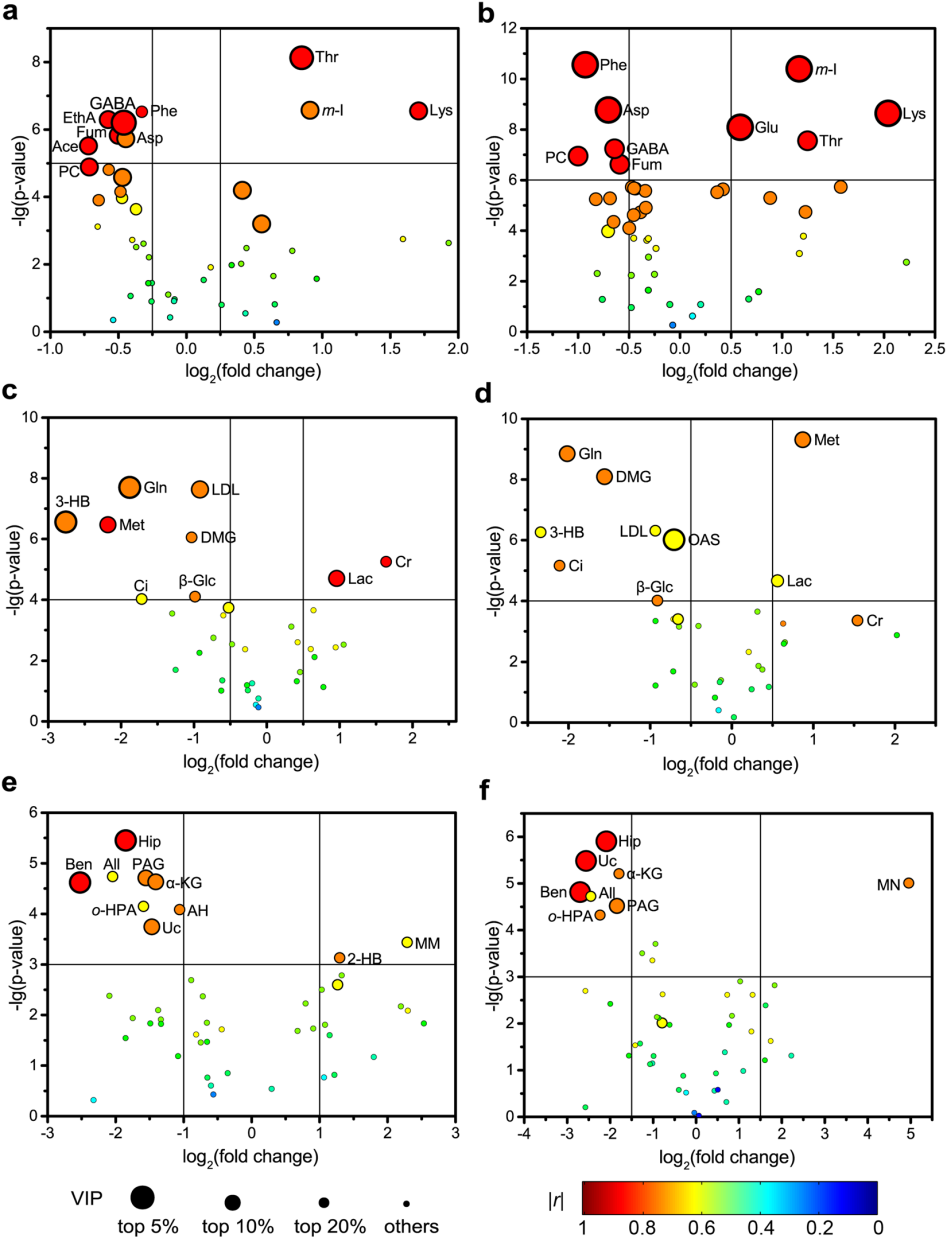
Enhanced volcano plots of multiple matrices for screening if metabolite markers. Figures (**a**,**c**,**e**) show comparison between SMFY and control groups, and (**b**,**d**,**f**) between GMFS and control groups; (**a** and **b**), (**c** and **d**), and (**e** and **f**) correspond to cortex extract, serum, and urine samples, respectively. VIP together with |*r*| is introduced with being represented by circles size and colour, respectively. For each comparison, VIP values are categorized into four segments: top 5%, top 10%, top 20% and rest 80%, with each represented by a circle of decreasing size.

The corresponding coefficient loading plots and enhanced volcano plots were then used to identify candidate metabolites that contributed to the inter-group separation. In the enhanced volcano plots, the fold change was defined as the ratio of average concentration of a given metabolite between the EA-treated groups (SMFY or GMFS) and the control group. Therefore, the concentrations for those metabolites located at positive side of horizontal axis in Fig. 5 are higher in the EA-treated group, compared to the controls. Detailed cut-off values for parameters to select significantly changed metabolites in enhanced volcano plots are listed in Table S1. In the enhanced volcano plots, |*r*| ≥ 0.6 (absolute correlation coefficient) and VIP ≥ top 20% (variable importance in projection) were set as the criteria to select metabolites with statistically significant changes between different groups. The cut-off values for *p*-value and fold change are indicated by straight lines in each plot. Generally, candidate metabolites identified by multivariate statistical analyses tend to locate at the upper left or upper right zones of the enhanced volcano plot (segmented by horizontal and vertical threshold lines into six zones) in larger circle shapes and warmer colours.

In comparison with the cortex metabolome of the control group, metabolic alterations in the SMFY group include decreased concentrations of aspartate, phenylalanine, acetate, fumarate, *γ*-aminobutyrate (GABA), ethanolamine (EthA), and phosphocholine (PC), together with increased concentrations of threonine, lysine, and *myo*-inositol (Figs 5a and S4a). By contrast, the GMFS group shows significant decreased levels of aspartate, phenylalanine, fumarate, GABA, and PC, together with increased levels of threonine, lysine, *myo*-inositol, and glutamate in cortex (Figs 5b and S4b). Among the changes, eight metabolites were found to have consistent changes following acupuncture and were considered as non-specific metabolic responses to the EA stimulation regardless of the meridian, and several others (e.g., EthA, acetate, and glutamate) were considered as exclusive responses due to stimulations on different meridians.

A combination of the enhanced volcano plot with correlation coefficient and variable importance projection (VIP) from OPLS-DA model offers a comprehensive and straightforward method to study differential metabolites. The results from enhanced volcano plots are consistent with the corresponding coefficient loading plots where metabolites with major difference between groups appear in hot colours.

Following EA stimulations, the serum samples of EA-treated groups (i.e., SMFY and GMFS) are highlighted with significant increases of lactate, along with reductions of glutamine, citrate, 3-hydroxybutyrate (3-HB), *β*-glucose, ***N*** *,* ***N***-dimethylglycine (DMG), and low density lipoprotein (LDL) in comparison with the control group (Fig. 5c and d). In addition, urinary metabolome analyses indicated significant lower levels of hippurate, urocanate, benzoate, allantoin, *α*-ketoglutarate (*α*-KG), phenylacetylglycine (PAG), and *ortho*-hydroxyphenylacetate (*o*-HPA) in both the SMFY and GMFS groups compared to the control group (Fig. 5e and f). Notably, the altered metabolites due to EA stimulations in blood and urinary samples are mainly scattered in the upper left region of the volcano plot, showing that EA stimulations primarily led to decreased metabolite concentration in serum and urine samples. In conclusion, these metabolites are potential non-meridian-specific markers due to EA stimulations.

The results also showed a number of meridian-specific markers due to EA treatment on the SMFY meridian, e.g., 2-hydroxybutyrate (2-HB, increase), creatine (Cr, increase), methylmalonate (MM, increase), aminohippurate (AH, decrease), acetate (Ace, decrease), and ethanolamine (EthA, decrease). On the other hand, treatment on the GMFS meridian is highlighted with meridian-specific changes, including increased concentrations of *N*-methylnicotinamide (MN) and glutamate, and accompanied by a reduction in *O*-acetylglycoprotein (OAS). Notably, methionine in blood serum was found to be decreased in the SMFY group but increased in the GMFS group (Fig. 5).

The stomach metabolome of the SMFY group does not show similarity with the GMFS group (shown in Fig. S5). These results support the distinctive effect of acupuncture at SMFY (the stomach meridian) acupoints on the stomach metabolome, different from acupuncture on the gallbladder meridian, GMFS. Since urine and serum reveal global metabolic variations, and cerebral-cortex-related neuro-regulation is of interest in this study, we primarily focused on the influences on urine, serum, and cortex tissue induced by acupuncture in following analyses.

### Pathway enrichment analysis

**Figure 6:**
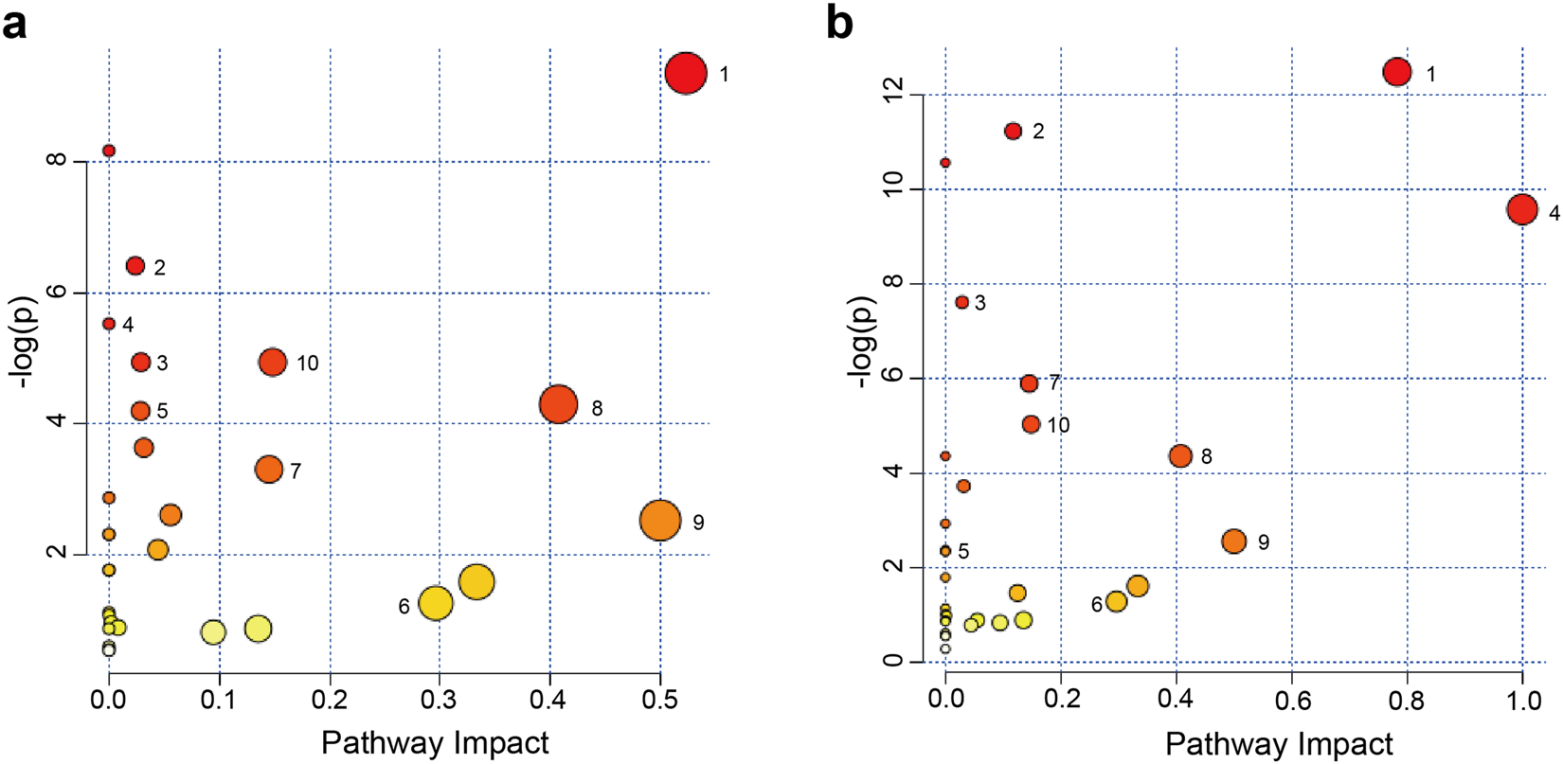
Bubble plots of altered metabolic pathways in multiple bio-samples (serum, urine, and cortex extract). (**a**) shows comparison between SMFY and control, and (**b**) between GMFS and control. Bubble area is proportional to the impact of each pathway with colour denoting the significance from highest (in red) to lowest (in white). 1, alanine, aspartate and glutamate metabolism; 2, arginine and proline metabolism; 3, butanoate metabolism; 4, D-glutamine and D-glutamate metabolism; 5, glycolysis or gluconeogenesis; 6, glyoxylate and dicarboxylate metabolism; 7, histidine metabolism; 8, phenylalanine metabolism; 9, phenylalanine, tyrosine and tryptophan biosynthesis; 10, TCA cycle.

A metabolic pathway analysis was conducted for serum, urine, and cortex extract samples using the MetPA (Metabolomics Pathway Analysis)^27^. The identified metabolic markers were analysed using the MetPA to facilitate further biological interpretation and thereby reveal the most relevant pathways involved in EA stimulations at acupoints on the SMFY or GMFS meridian. The most affected pathways in the experimental groups were found to be alanine, aspartate, and glutamate metabolism; phenylalanine metabolism; and phenylalanine, tyrosine and tryptophan biosynthesis (Impact value ≥ 0.4 and -log(*p*) ≥ 2) (Fig. 6 and Table S2). The EA stimulations on acupoints of SMFY and GMFS meridians share similar effects on a number of metabolic pathways; for example, metabolites in the TCA cycle were found changed in the same direction (but to a different degree) with EA treatment on either SMFY or GMFS acupoints. In contrast, marked increased metabolic changes in glycolysis can only be observed in the SMFY group, while glutamine and glutamate changed significantly only for the GMFY group.

### Statistical power analysis

**Figure 7:**
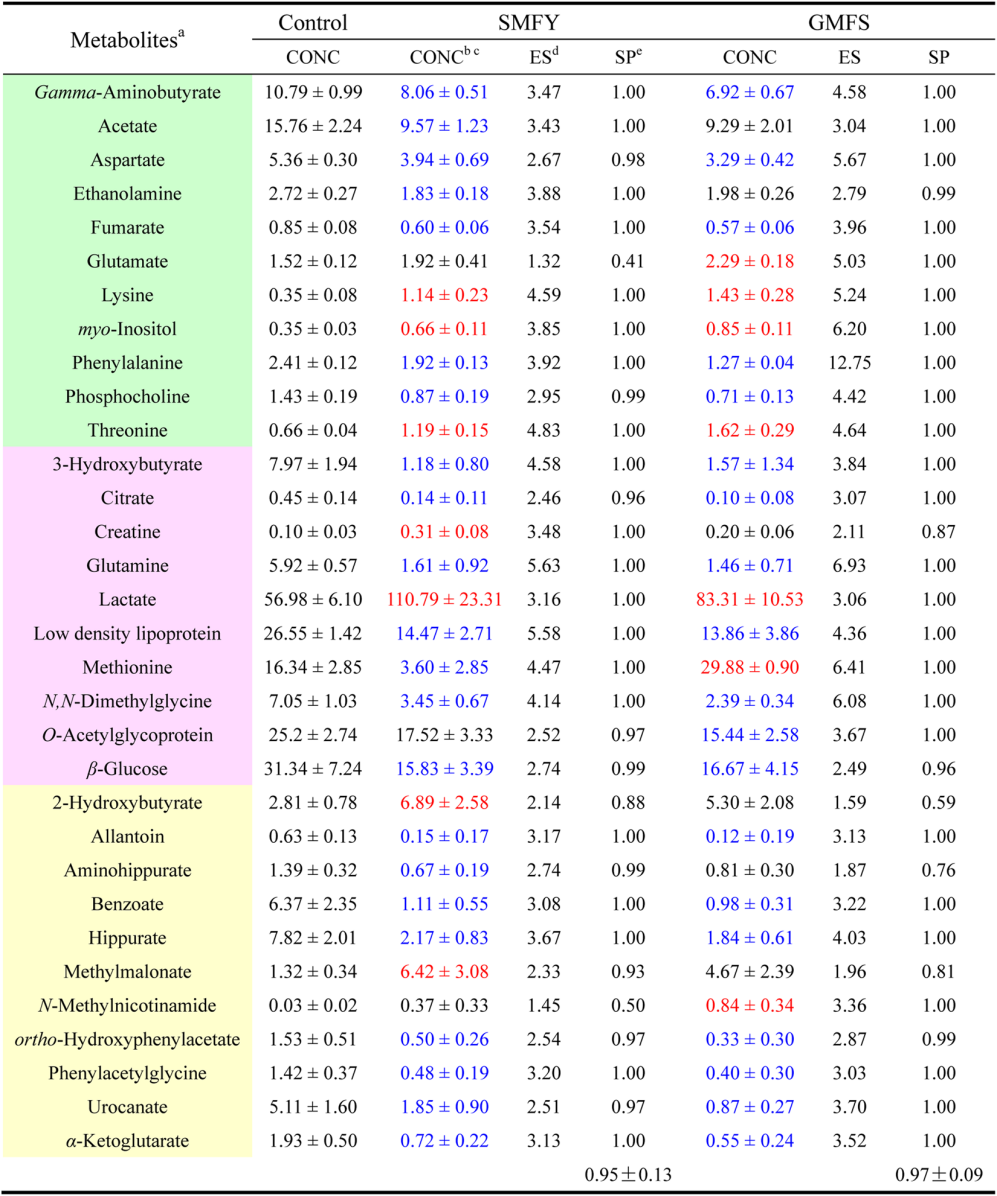
The post hoc power analysis of the selected metabolites calculated in G*power. (**a**) colour coded according to the different bio-samples,  (light green),  (soft red), and  (light yellow) present cortex extract, serum, and urine respectively. (**b**) The relative concentration (percentage of the normalized integrals, mean ± SD). (**c**) Red coloured numbers denote significant increase whereas the blue coloured ones indicate significant decrease and the black coloured ones denote no significant changes in the EA-treated group (i.e., SMFY and GMFS) compared to the control group. The criteria of selection are as shown in Table S1. (**d**) The effect size computed based on the standardized mean difference between the EA-treated group (i.e., SMFY and GMFS) and the control group in G*power. (**e**) The statistical power calculated as a function of critical significance level (*α* = 0.01), given sample size (*n* = 8), and obtained variable effect size in G*power.

To determine the achieved statistical power of our study, a post hoc power analysis was carried out on the current results using the online statistics software G*power 3.1^28, 29^ (http://www.gpower.hhu.de/). First, the effect size was computed as the absolute difference between the experimental mean and the control mean divided by a standard deviation for the NMR bio-sample data. The resultant large effect sizes indicated the sufficient difference between the experimental groups and the control group (Fig. 7). These variable effect sizes were then used to calculate the achieved statistical power of the selected metabolites as a function of specified values for critical significance level (*α* = 0.01) and given sample size (n = 8) (refer to Fig. S6 for test specification). The statistical power for all significant metabolites were found to be greater than 0.8 (Fig. 7). The means statistical power of 0.95 (SD = 0.13) and 0.97 (SD = 0.09) were obtained for the SMFY group and the GMFS group, respectively. The great effect size and achieved statistical power establish the reliability of our results.

## Discussion

**Figure 8:**
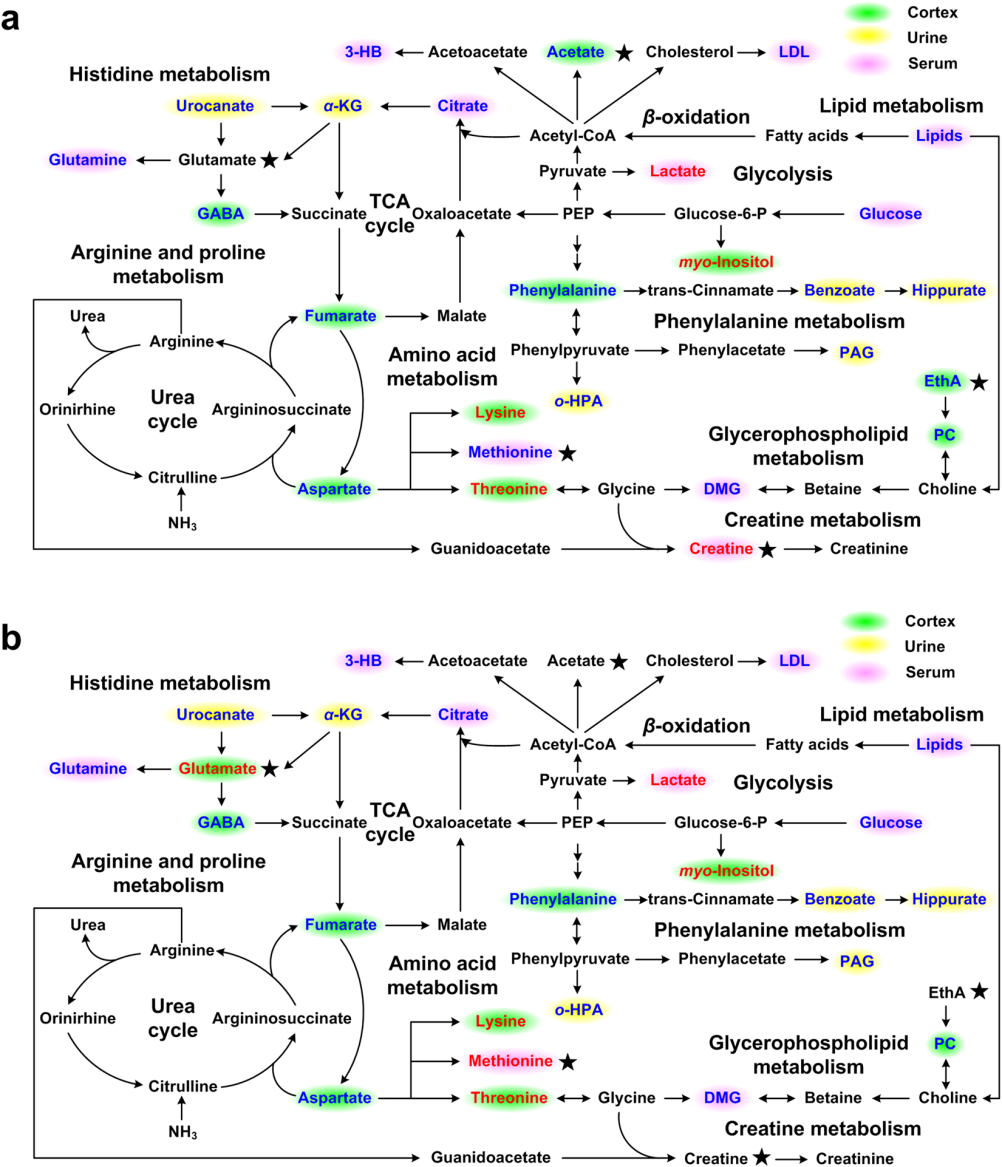
Summaries of metabolic pathways altered after EA stimulations. (**a**) delineates SMFY group, and (**b**) GMFS group. Metabolites displayed with red (or blue) present concentration significantly increases (or decreases) in comparison with the control group; those in black indicate no significant changes; the metabolites with ★ indicate exclusive metabolites changes for SMFY or GMFS groups. PEP, Phosphoenolpyruvate.

The experimental results showed a number of common metabolic changes caused by EA stimulation as well as specific metabolic perturbations due to EA treatment on either the SMFY or GMFS meridians. These EA-induced metabolic changes primarily involved metabolites in the amino acid metabolism, energy metabolism, fatty acid *β*-oxidation, choline metabolism and gut microbiota-related metabolism (Fig. 8). These pathways are affected to different extents based on results from pathway analysis (Fig. 6). The current results suggested that meridian-specific metabolic changes can be detected using a NMR-based metabolomics approach.

In this study, we found that GABA concentration was lowered by EA stimulations relative to the control group. GABA is a key mediator of inhibitory neurotransmission in mammalian central nervous system (CNS)^30^. It can couple and activate GABA_A_ and GABA_B_ receptors to reduce brain anxiety. Known to exert an antagonistic effect upon excitatory neurotransmitters, such as dopamine and glutamate, GABA can be synthesized from glutamate via glutamate decarboxylase. Mutual transformations among glutamine, glutamate (excitatory), and GABA (inhibitory) maintain the internal balance between excitation and inhibition in the CNS^31^. The decreased levels of GABA and glutamine in both SMFY and GMFS groups indicated that EA may partially reduce the inhibition of excitement in the rat brain. With the development of neuroimaging techniques, the use of positron emission tomography (PET) and functional magnetic resonance imaging (MRI) to explore the central mechanism of acupuncture has been an active area of research^32^. Notably, functional MRI studies report that EA stimulation on SMFY and GMFS acupoints contribute to activation of brain regions^33–36^. Recent papers have described the central regulations over the benign and comprehensive regulatory effects of acupuncture by brain, and certain findings are similar to the results of the current study. Zeng have suggested that acupuncture stimulation not only affect the activity of the common pathway of somatic and visceral sensation but also modulate the activity of certain brain functional regions^37^. Accordingly, our study further found neurotransmitter (such as GABA, glutamate) changes by EA stimulation on the SMFY or GMFS acupoints. Moreover, EA at SMFY acupoints or GMFS acupoints can enhance CNS excitement significantly, with the GMFS group exhibiting more prominent changes. The brain seems to play an active role to convey the effect of acupuncture treatment.

Altered levels of glucose, lactate, fumarate, citrate, *α*-KG, and ATP in the present study suggested that the EA stimulations induced the change of energy metabolism. As intermediates of the TCA cycle, fumarate, citrate, and *α*-KG decreased in SMFY and GMFS groups in comparison with the control group, unveiling the enhanced aerobic TCA cycle by EA stimulations (Fig. 6).

From the above analyses, significant changes of neurotransmitter (GABA, glutamate) levels and increased energy metabolism may affect the catabolism of amino acids. Phenylalanine acts as a precursor for the biosynthesis of catecholamine (i.e., dopamine, epinephrine, and norepinephrine)^19^. In comparison with the control group, the observed decreased levels of phenylalanine in the SMFY and GMFS groups were probably related to demands for catecholamine biosynthesis, which will lead to excitation of central nerves. Lysine is a structural component of carnitine, which is capable of increasing fatty acid *β*-oxidation^38^. High levels of lysine in the SMFY and GMFS groups may be associated with the slow oxidization of fatty acids into acetyl-CoA after EA stimulations. This can be reflected by the decreased level of 3-hydroxybutyrate (3-HB), which is a final product and marker of fatty acid *β*-oxidation in mitochondria (Fig. 8). The phosphatide methylation of the liver cell membrane will enhance the membrane mobility to prevent bile deposition in liver cells and then promote detoxification, thus providing liver protection with methionine. The GMFS meridian is tightly linked to liver function, and EA at the GMFY acupoints will effectively accelerate bile release to facilitate the normal physiological function of liver cells. Because EA stimulations directly accelerate bile release, the dependence on methionine fades, eliciting the accumulation of methionine in the GMFS group (higher level in comparison with the control group).

Abnormalities in lipid metabolism, including significantly elevated LDL/VLDL and cholesterol, have been observed in the serum of normal weight migraine patients^39^ and pregnant migraineurs^40^. In our study, serum LDL in both the SMFY and GMFS group were dramatically decreased, with an approximately 0.9-fold change in comparison with the control group, revealing that EA at SMFY acupoints and GMFS acupoints may lower cholesterol and slow the conversion from cholesterol to LDL and therefore help to provide potential prevention and treatment for cardiovascular diseases such as hypercholesterolemia and atherosclerosis. Furthermore, an increased severity of liver fibrosis has been reported to be associated with higher tyrosine, phenylalanine, methionine, citrate, and LDL^41^. EA stimulations at the SMFY and GMFS acupoints can lower phenylalanine, LDL, and citrate levels, resulting in an increased capacity for citrate clearance in cirrhotic patients and a lower rate of lipogenesis.

Hippurate, benzoate, and urocanate are common metabolites produced by gut microbiota. Significant decreased levels of those urinary metabolites were observed in the SMFY and GMFS groups, indicating that EA stimulations at SMFY and GMFS acupoints alter the metabolism of gut microbiota in the gastrointestinal tract. This is consistent with the practical EA applications that SMFY is generally selected as the primary meridian with GMFY as the complementary approach for treatment of gastrointestinal diseases^42, 43^.

Accumulating research shows an interconnection between the SMFY meridian and the stomach. In an acupuncture study on zusanli (ST36) acupoint in the SMFY meridian, Zhang utilized bioinformatics analysis to determine the molecular function of differentially expressed proteins^23^; the most interesting functional categories were “gastric acid secretion” and “pancreatic secretion”, representing 2.53% and 6.33% of all the proteins identified, respectively. The result would help to explain why the ST36 point has been traditionally used in human acupuncture to treat gastrointestinal disorders. After comprehensive analyses of the metabolic markers, we found that EA treatment on SMFY meridian seems to promote glycolysis. Therefore, EA treatments can be adopted to enhance the energy supplement under pathologies and thus facilitate the physical recovery. Previous studies^44, 45^ have shown that the effects of EA at acupoints of SMFY on gastric motility were related to brain-gut peptides. Therefore, our study provides supporting evidence that brain metabolism plays an important role in acupuncture treatment.

In this study, we chose healthy rats with no EA treatment as controls instead of using controls with EA on non-acupoints near to the studied acupoints as the baseline to determine metabolic variations. This experimental design aimed to observe the maximum possible EA-induced metabolic variations, since it has been reported that EA on near-acupoint regions will also introduce certain metabolic variations similar to EA on acupoints. In other words, acupoint-specific metabolic variation will decrease with the distance between EA point and actual acupoint, thus reducing the contrast between the EA group and control group. Moreover, acupoints for some meridians are close to each other, and EA on near-acupoint region may introduce metabolic variation originating from a different acupoint.

The results and conclusions of the current study should be cautiously interpreted due to the following limitations. First, meridian specificity consists of different aspects, e.g., biophysics specificity, dynamic temporal specificity, spatial specificity, and viscera-related specificity^17, 46, 47^. Therefore, future studies may be focused on these aspects to provide an in-depth systematic understanding of meridian specificity. Second, although the sufficient statistical power on the sample size (n = 8) was tested using software G*power (http://www.gpower.hhu.de/), further study with a larger sample size is recommended to validate the current conclusions. Third, future study could include female rats to examine the interaction between gender effect and acupuncture treatment. Finally, the current study applied a single metabolomic platform (NMR) and therefore does not cover a wide range of metabolomes due to the diversity of physicochemical properties and the broad range of metabolite concentrations. Thus, future study with multiple metabolomics platforms (e.g., GC-MS and LC-MS) or even multiple omics technologies (e.g., proteomics and transciptomics) can be integrated to provide more comprehensive and definitive meridian-specific markers^48–50^.

In conclusion, we performed EA stimulations on the acupoints of SMFY and GMFS meridians to investigate meridian specificity. The ^1^H NMR-based metabolomics approach was adopted to identify differential rat metabolic profiles of multiple biological matrices (i.e., serum, urine, stomach and cortex extracts) induced by EA stimulations. Our current study demonstrated significant metabolic pattern differences in response to EA stimulations on the acupoints of the SMFY and GMFS meridians. These results demonstrate the feasibility and effectiveness of the metabolomics approach in understanding the effects of acupuncture, provide a metabolic basis for meridian specificity in acupuncture treatments, and constitute a reference for the clinical practice of acupuncture.

## Methods

### Ethical Statement

All animal procedures were approved and conducted in strict accordance with the guidelines of the Animal Care and Use Committee of Hunan University of Chinese Medicine (Permit Number: SCXK2016–0015). The study was carried out adhering to guidelines provided by the National Institutes of Health for the Care and Use of Laboratory Animals and all efforts were made to minimize the suffering of animals.

### Animals and housing

*Sprague Dawley* (SD) rats in a single gender (male) were used in our experiment. It should be noted that female rats generally show higher metabolic variation due to differences in hormonal profiles, food intake and energy metabolism at different stages of the oestrous cycle. To prevent metabolic interference due to gender factors, we only used male rats in the current study. Briefly, 24 healthy male SD rats (150 ± 20 g, 8-weeks-old) were individually housed in metabolic cages under controlled conditions (temperature, humidity, and 12-h light/dark cycle). Food and water were available *ad libitum*. After a one-week acclimation, all 24 rats were randomly divided into three groups (*n* = 8): control group (without EA treatments), SMFY group (EA at SMFY acupoints), and GMFS group (EA at GMFS acupoints).

The sample size used in the current study was calculated based on our preliminary study before the current experiment. Briefly, we conducted a Power Analysis using MetaboAnalyst 3.0 software (http://www.metaboanalyst.ca) to analyse rat blood serum data (with FDR = 0.13, power = 0.8). It was found that n = 8 is adequate to meet the requirements (Fig. S7).

### EA stimulations

For the SMFY group, three classical acupoints along SMFY meridian (including *Sibai* (ST 2), *Liangmen* (ST 21), and *Zusanli* (ST 36), represent acupoints of head, trunk, and limb, respectively) were selected for EA stimulations. *Yangbai* (GB 14), *Riyue* (GB 24), and *Yanglingquan* (GB 34) in the same segments along the GMFS meridian were selected for EA treatment for the GMFS group (shown in Fig. S8). Locations for both SMFY and GMFS acupoints were determined according to Government Channel and Points Standard GB12346–90 of China and “The Veterinary Acupuncture of China”. State-licensed acupuncturists performing all treatment procedures had at least 2 years of experience with acupuncture treatment. For acupuncture treatment, two-channel electrical stimulations were performed with a pulse generator (Model G6805-II; Qingdao Xinsheng Medical Instrument Factory, Shandong, China) with four sterile stainless-steel acupuncture needles (diameter: 0.25 mm) being inserted into the acupoints. The electrical stimuli consisted of both intermittent and irregular waves (intermittent wave: 4 Hz, irregular wave: 50 Hz) with voltages ranging from 2 to 4 V. Electrical intensities were increased from 0.1 mA to 1.0 mA until the rats’ hind limbs began to twitch slightly. Rats of the SMFY and GMFS groups were given EA stimulations for 30 minutes per day for a consecutive seven days.

### Sample collections

After the seven-day treatment course, 24-hour urine samples for each of the rats were collected into 5 mL Eppendorf (EP) tubes on ice. Each of the tubes contained a drop of NaN_3_ solution (0.1 g/mL NaN_3_). In addition, blood samples (~1 mL) drawn from rats’ carotid arteries were collected into 5 mL EP tubes with no anticoagulation and left to clot at room temperature for 20 min. The supernatants (serum) were obtained by centrifugation at 11,000 × ${\rm{g}}$, at 4 °C for 10 min. Finally, the rats were sacrificed by rapid decapitation without anaesthesia in 30 min or less; the specimens of cortex and stomach tissues were then dissected immediately (typically within 30 s), and snap-frozen in liquid nitrogen. All biological samples were stored at −80 °C until further treatments.

### Sample preparations for NMR analysis

For urinary samples, 300 μL urine was added with 300 μL phosphate buffer solution (1.5 M K_2_HPO_4_/NaH_2_PO_4_, pH 7.4, 99.9% D_2_O with 0.3 mM TSP (3-trimethylsilyl- propionic-2,2,3,3-d4 acid sodium salt). The D_2_O provided the NMR spectrometer with a field frequency for locking, and TSP was used as a reference for chemical shift (0 ppm). After centrifugation (11,000 × ${\rm{g}}$, 4 °C, 10 min), 500 μL supernatants were transferred into 5-mm NMR tubes. For blood serum samples, an aliquot of 400 μL was added with 200 μL phosphate buffer solution (90 mM K_2_HPO_4_/NaH_2_PO_4_, pH 7.4, 99.9% D_2_O). After centrifugation (11,000 × ${\rm{g}}$, 4 °C, 10 min) to remove precipitates, 500 μL supernatants were transferred into 5 mm NMR tubes.

The frozen excisions of cortex and stomach tissues (~300 mg) were ground on dry ice using a mortar and pestle, then transferred into micro-centrifuge tubes with adding an icy cold solvent mixture of methanol and water (2:1, v/v). After sonication for 15 min in a water bath, the resulting homogenates were added with chloroform and water (1:1, v/v), placed on ice for 15 min, and then centrifuged (11,000 × ${\rm{g}}$, 4 °C, 10 min). Supernatants (upper aqueous phases) were collected into 5 mL EP tubes and lyophilized under liquid nitrogen for approximately 36 h to remove methanol. The dried residues were re-dissolved with a 550 μL phosphate buffer solution (90 mM K_2_HPO_4_/NaH_2_PO_4_, pH 7.4, 99.9% D_2_O with 0.3 mM TSP). Following vortex and centrifugation (11,000 × ${\rm{g}}$, 4 °C, 10 min), 500 μL supernatants (tissue extracts) were pipetted into 5 mm NMR tubes. All prepared biological samples (i.e., serum, urine, cortex, and stomach) in NMR tubes were stored at 4 °C until NMR analyses.

### NMR experiments

^1^H NMR spectra of serum, urine, cortex, and stomach were acquired using a 600 MHz Bruker NMR system at 298 K. Serum samples were analysed by the Carr-Purcell-Meiboom-Gill (CPMG) sequence (awaiting time ~ π/2 ~ [τ ~ π ~ τ]_n_ ~ acquisition) with a free relaxation duration (2nτ) of 100 ms and an echo time (τ) of 250 μs. For urine, cortex and stomach samples, Nuclear Overhauser Effect Spectroscopy (NOESY, awaiting time ~ π/2 ~ t_1_ ~ π/2 ~ t_m_ ~ π/2 ~ acquisition) was used with a water suppression lasting 2 s and a mixing time (t_m_) of 120 ms. For data acquisition, 64 scans were performed with 32 k data points under a spectral width of 12,000 Hz for each free induction decay (FID).

All ^1^H NMR spectra were manually phased and baseline corrected using MestReNova v.8.1.2 software (Mestrelab Research S.L.). TSP (at δ 0.00) was used as a spectral reference for urine, stomach and cortex, and the left split (-CH_3_) from the doublet of lactate centring at δ 1.336 (methyl group) was used as chemical shift reference for serum samples. Residual water signals (serum: δ 4.7–5.0, urine: δ 4.76–5.06, cortex: δ 4.97–5.02 and stomach: δ 4.95–5.04), urea resonances (δ 5.60–6.35), and peak-free regions were excluded from further analysis. The remaining spectra over ranges of δ 0.5–9.0 for serum, δ 0.5–10.0 for urine, δ 0.5–8.5 for cortex and δ 0.5–9.2 for stomach were binned into bucketed data with a fixed width of 0.004 ppm (2.4 Hz). Prior to multivariate data analysis, data normalization was carried out using the method of probabilistic quotient normalization (PQN)^51^ to take the sample-concentration variations into considerations.

### Multivariate statistical data analyses

The pre-processed bucketed data were imported into the SIMCA-P software (version 12.0.1, Umetrics AB, Ume°a, Sweden) for multivariate analyses. The normalized bucket data were scaled by mean centre (Ctr) and subjected to principal components analysis (PCA) to overview the data distribution and potential outliers. Supervised partial least squares-discriminate analysis (PLS-DA) and orthogonal partial least squares discrimination analysis (OPLS-DA) were then implemented on Pareto scaled NMR data to examine metabolite differences between groups. The validation of the model was performed with a 7-fold cross-validation and permutation test (200 permutations)^52^. The loading plots from OPLS-DA models were generated with an in-house MATLAB program, and signals were colour-encoded with correlation coefficients to exhibit metabolites with significant changes.

Statistical analyses were also performed using methods of fold-change and the Student’s *t*-test with a Bonferroni correction. The resulting *t* statistic, such as transformed *p*-value, can be used to determine metabolites with statistically significant changes in metabolomics. The fold-change is a method to evaluate the log ratio of concentrations between two conditions to identify significant metabolite variations above an arbitrary cut-off value. The metabolites were quantified by integrals over corresponding spectral range in reference to the internal standards. To avoid the influences induced by spectral congestion, we chose peaks with least overlapping for quantifications for corresponding metabolites of interest.

In this study, we used the volcano plot to summarize both *t*-test and fold-change criteria in a single plot. Typically, it is a scatter plot of -log_10_(*p*-value) against log_2_(fold-change)^53^. The variable importance projection (VIP) and absolute correlation coefficient values (|*r*|) constructed from the OPLS-DA analysis were introduced as two new variables in the original volcano plot and were represented by circles size and colour, respectively (i.e., larger circle size corresponds to larger VIP value, warmer colour to higher |*r*|). This enhanced four-dimensional volcano plot may provide an integrated and effective method to identify potential biomarker candidates from a global view.

## Electronic supplementary material

Supplementary Information
