# Supplementary material for: 1H NMR-based Investigation of Metabolic Response to Electro-Acupuncture Stimulation
Source: Sci Rep. 2017 Jul 28;7:6820. doi: 10.1038/s41598-017-07306-5 (PMC5533752; doi:10.1038/s41598-017-07306-5)
Supplement: Supplementary file 1 — Supplementary Information [file 41598_2017_7306_MOESM1_ESM.pdf]

Supplementary Information

^{1}H NMR-based Investigation of Metabolic Response to Electro-Acupuncture Stimulation

Caigui Lin[1], Zhiliang Wei[2], Kian-Kai Cheng[3], Jingjing Xu[1], Guiping Shen[1], Chang She[4], Huan Zhong[4], Xiaorong Chang[4*] & Jiyang Dong[1*]

1. Department of Electronic Science, Xiamen University, Xiamen 361005, China;

2. Department of Radiology, The Johns Hopkins University, Baltimore, Maryland 21205, USA;

3. Department of Bioprocess & Polymer Engineering and Innovation Centre in Agritechnology, Universiti Teknologi Malaysia, Johor 81310, Malaysia;

4. College of Acupuncture & Moxibustion and Tui-na, Hunan University of Chinese Medicine, Changsha 410007, China.

Submitted to **Scientific Reports**

*Corresponding authors: J. Dong (jydong@xmu.edu.cn) and X. Chang (xrchang1956@163.com)

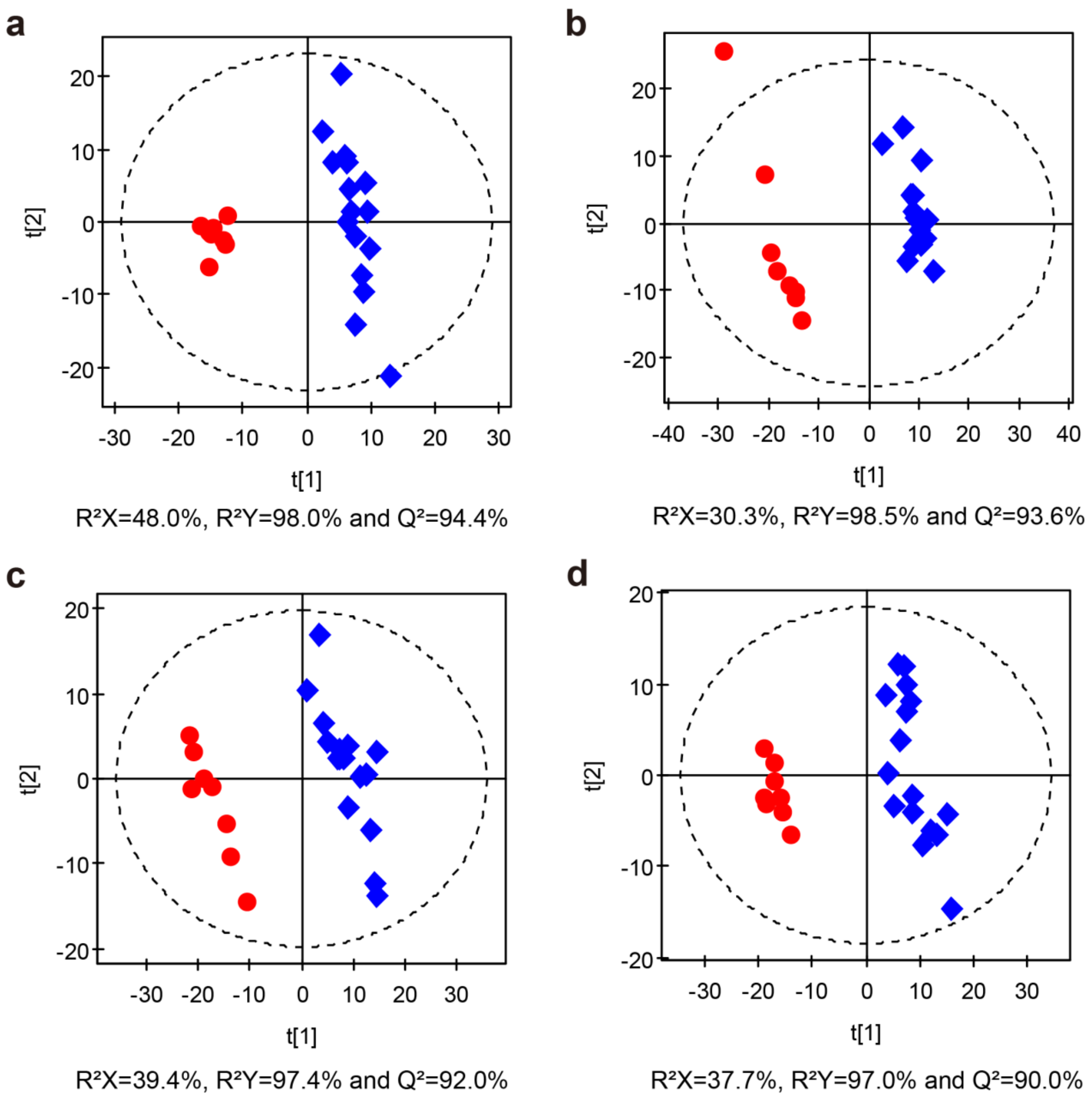

Fig. S1 PLS-DA scores plots of control (●) and EA-treated (◆) groups for (a) serum, (b) urine, (c) stomach extract, and (d) cortex extract.

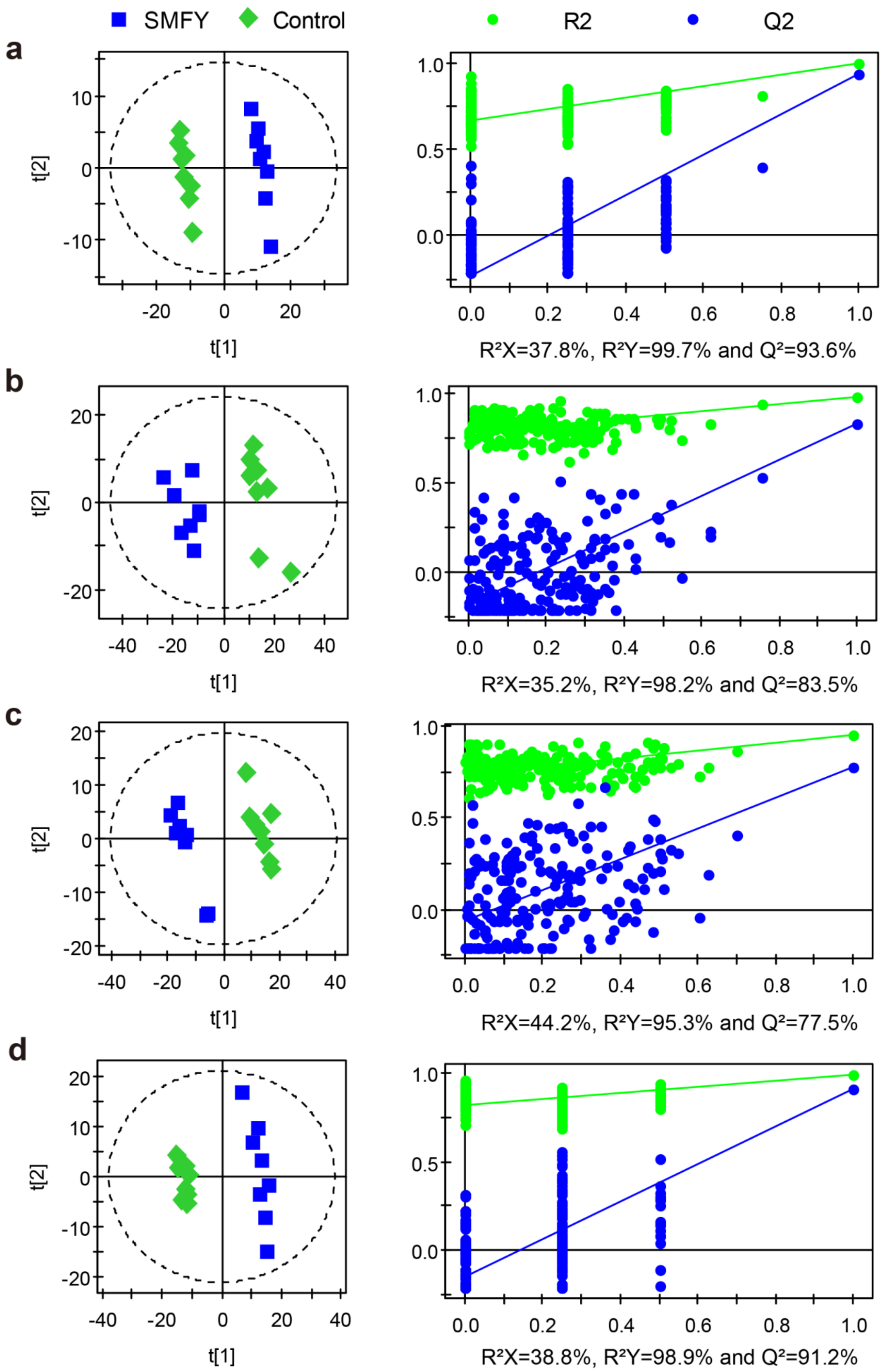

Fig. S2 PLS-DA scores plot (left) and the corresponding validation plot (right) between the SMFY and control groups. (a) serum, (b) urine, (c) stomach extract, and (d) cortex extract.

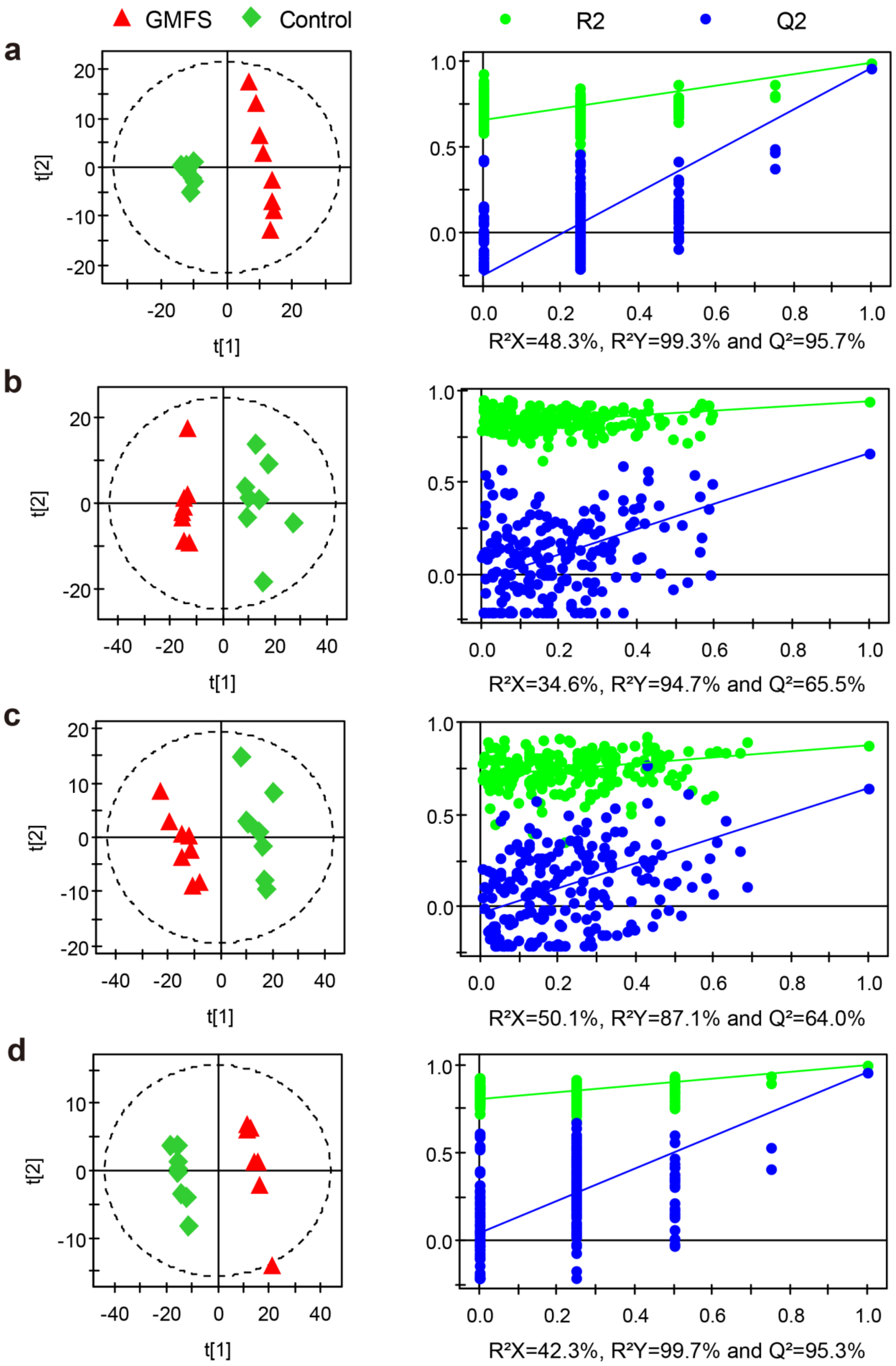

Fig. S3 PLS-DA scores plot (left) and the corresponding validation plot (right) between the GMFS and control groups. (a) serum, (b) urine, (c) stomach extract, and (d) cortex extract.

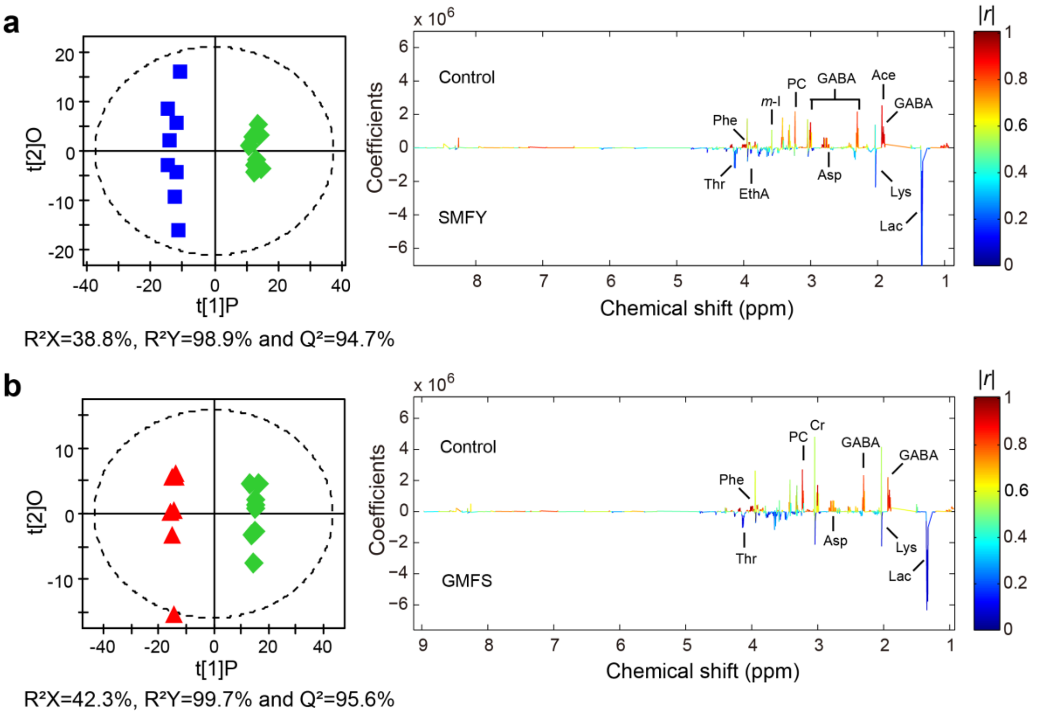

Fig. S4 OPLS-DA scores plot (left) and the corresponding loading plot with correlation coefficient color coded (right) from extracted cortex NMR data. The symbols ◆(green filled rhombus), ■(blue filled square), ▲(red filled triangle) represent the control, SMFY, and GMFS groups, respectively. Figure (a) presents comparison between the SMFY and control groups and (b) between the GMFS and control groups.

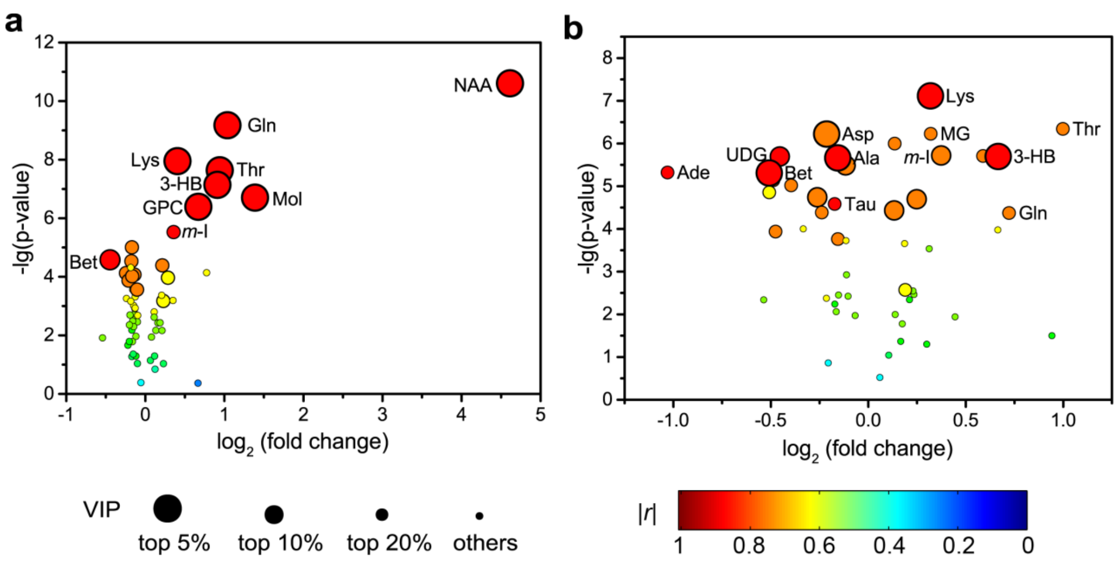

Fig. S5 Enhanced volcano plots of stomach extracts for screening metabolite markers. Figure (a) shows comparison between SMFY and control groups, and (b) between GMFS and control groups. VIP together with |*r*| is introduced with being represented by circles size and color, respectively. For each comparison, VIP values are categorized into four segments: top 5%, top 10%, top 20% and rest 80% with each represented by circle of decreasing size.

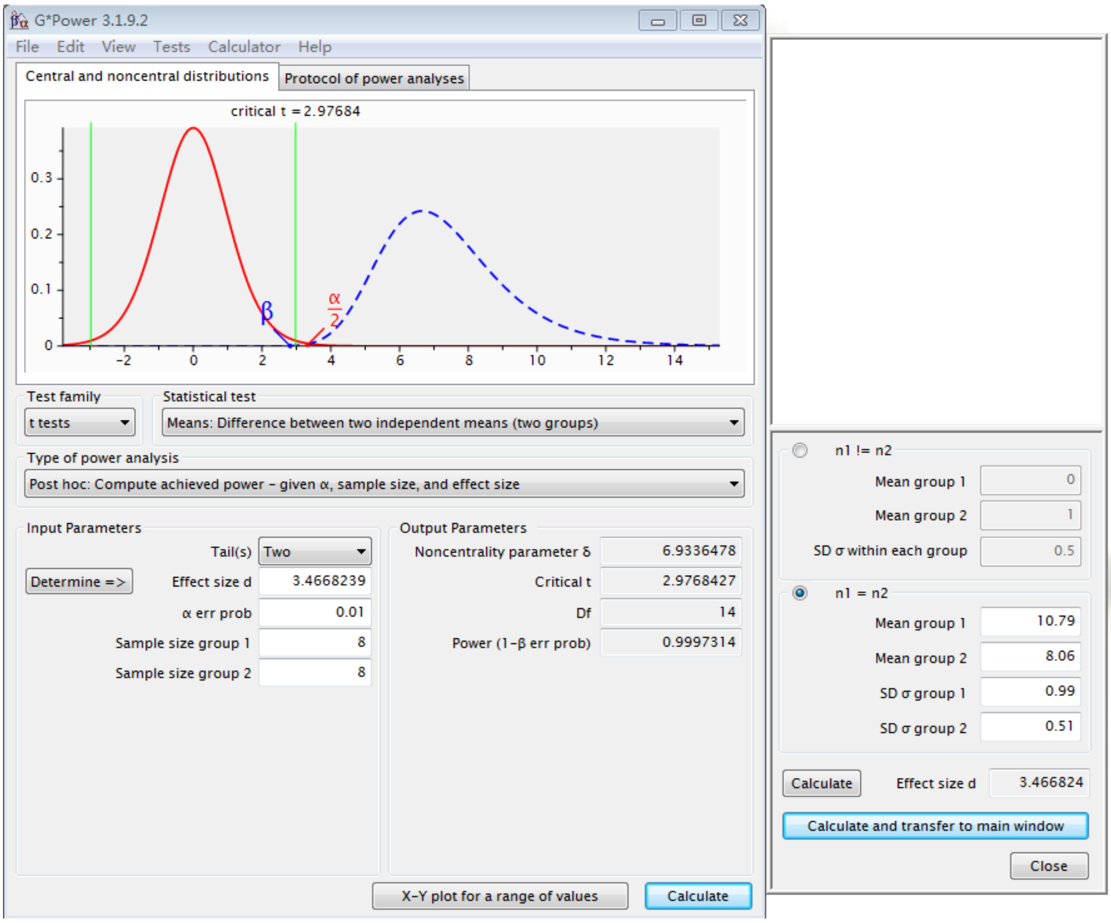

Fig. S6 The main window of the post hoc power analysis specification in Gpower 3.1 and the "Effect size" drawer. Take the *Gamma*-Aminobutyrate selected between the SMFY and the control groups as an example.

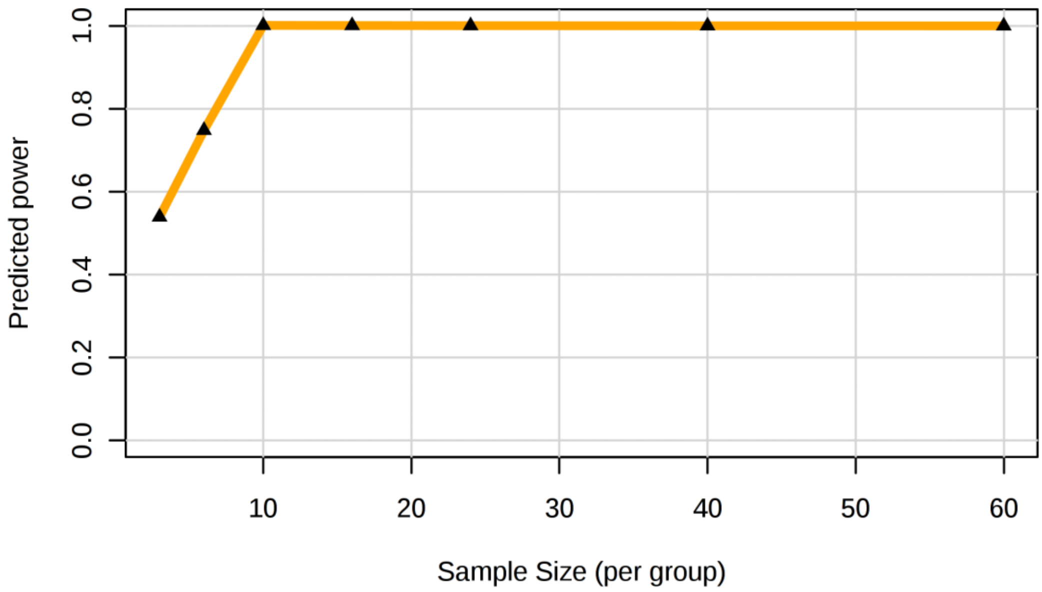

Fig. S7 Sample size profile for serum data with FDR=0.13.

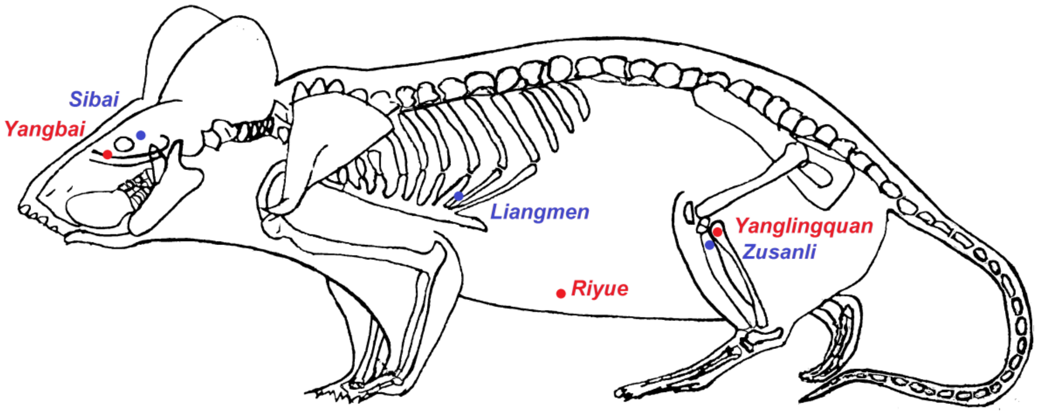

Fig. S8 Location of the SMFY (blue dot) and GMFS (red dot) acupoints (which was re-drawn by Dr Jingjing Xu). The acupuncture points were located as follows: (i) ST-36 (*Zusanli*), approximately 1 mm lateral to the tibial tuberosity; (ii) GB-34 (*Yanglingquan*), approximately 3 mm upper lateral to ST-36 (*Zusanli*); (iii) ST-2 (*Sibai*), approximately 5 mm above the orbit; (iv) GB-14 (*Yangbai*), approximately 5 mm under the orbit; (v) ST-21 (*Liangmen*), approximately 3 mm above the pectoral suferficialis muscle; (vi) GB-24 (*Riyue*), approximately 20 mm above the navel.

Table S1 Cutoff value for significant metabolites selection relative to the control group.

Parameter	Cortex		Serum		Urine	
	SMFY	GMFS	SMFY	GMFS	SMFY	GMFS
VIP[a]	Top 20%	Top 20%	Top 20%	Top 20%	Top 20%	Top 20%
$\|r\|$[b]	0.6	0.6	0.6	0.6	0.6	0.6
p-value[c]	1×10^{-5}	1×10^{-6}	1×10^{-4}	1×10^{-4}	1×10^{-3}	1×10^{-3}
$\log_2$(fold change)[d]	±0.25	±0.50	±0.50	±0.50	±1.00	±1.50

[a] VIP: the variable importance projection constructed from the OPLS-DA analysis.

[b] $|r|$: the absolute correlation coefficient values constructed from the OPLS-DA analysis.

[c] *p*-value: obtained from the Student's *t*-test against the control group.

[d] $\log_2$(fold change): the original fold change calculated from the logarithm analysis, the fold change was defined as ratio of average concentration of a given metabolite between the experimental groups (SMFY or GMFS) and the control group.

Table S2 Ingenious pathway analysis result for metabolomics data.

Pathway name	Total[a]	SMFY group			GMFS group		
		Hit[b]	Impact[c]	-log(p)	Hit	Impact	-log(p)
Alanine, aspartate and glutamate metabolism	24	5	0.52	9.37	6	0.78	12.48
Arginine and proline metabolism	44	5	0.02	6.42	7	0.12	11.23
Butanoate metabolism	20	3	0.03	4.94	4	0.03	7.61
D-glutamine and D-glutamate metabolism	5	2	0.00	5.53	3	1.00	9.57
Glycolysis or Gluconeogenesis	26	3	0.03	4.20	2	0.00	2.37
Glyoxylate and dicarboxylate metabolism	16	1	0.30	1.25	1	0.30	1.28
Histidine metabolism	15	2	0.15	3.30	3	0.15	5.89
Phenylalanine metabolism	9	2	0.41	4.30	2	0.41	4.37
Phenylalanine, tyrosine and tryptophan biosynthesis	4	1	0.50	2.52	1	0.50	2.56
TCA cycle	20	3	0.15	4.94	3	0.15	5.04

[a] Total: the total number of compounds in the pathway.

[b] Hits: the actual matched number from the user uploaded data.

[c] Impact: the pathway impact value calculated from pathway topology analysis.

[d] -log (*p*): the original *p* value calculated from the logarithm analysis.

Supplementary method for Pathway enrichment analysis

For Pathway enrichment analysis in the current study, we had chosen pathways with criteria of Impact value ≥ 0.4 and -log(p) ≥ 2. In statistics, significance level of p ≤ 0.01 is commonly used. Therefore, we had used -log(p) ≥ 2 as the threshold for the Pathway enrichment analysis.

On the other hand, impact value is a measure of topological importance of a pathway. According to the KEGG pathway database, there are a total of 84 pathways for rats. In the current study, metabolites from 46 pathways were detected by the NMR analysis. Therefore, we had analyzed the impact values of these 46 metabolic pathways and obtained the following results: For the comparison between the GMFS group and control group, the mean of impact values was found to be 0.085. In addition, the comparison between the SMFY group and the control group, the mean of impact values was 0.056. Previously, many topological characters of complex network (*e.g.* the out-degree of nodes) were found to be subjected to power-law distribution (S.H. Stogatz. Exploring complex networks. ***Nature***, 410: 268-276, **2001**). Similarly in the current study, we had assumed that the impact values of pathways to be subjected to a power-law distribution as follows,

$$f(x) = \begin{cases} \lambda e^{-\lambda x} & x > 0 \\ 0 & x \ll 0 \end{cases}. \qquad (1)$$

The experimental frequency distribution (blue bars) and theoretical probability density distribution (red curves) of the impact values are shown in the following figures. **Fig. S9** demonstrates that the impact values of pathways are subject to power-law distribution Eq.(1).

By using the distribution function with $\lambda = mean$ and $p \leq 0.01$, the pathway is significantly different for impact value ≥ 0.4. Therefore, we had chosen impact value ≥ 0.4 as an additional threshold for Pathway enrichment analysis.

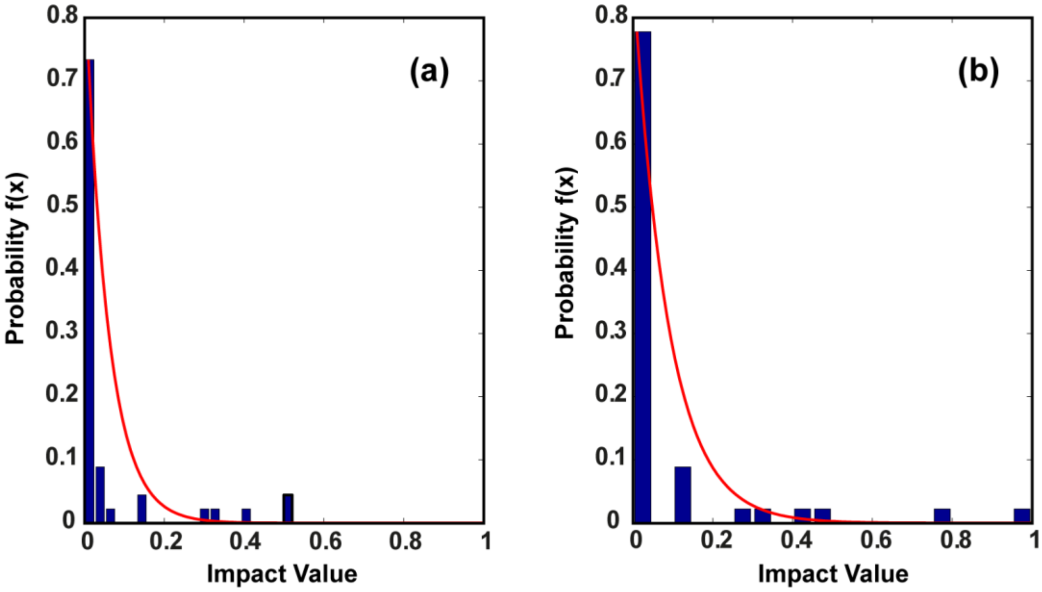

Fig. S9 The frequency distribution (blue bars) and probability density distribution (red curves) of the impact values of pathways for (a) SMFY sub-networks ($\lambda = 0.056$) , and (b) GMFS sub-networks ($\lambda = 0.085$).
